# Deposition of metal particles onto semiconductor nanorods using an ionic liquid

**DOI:** 10.3762/bjnano.10.71

**Published:** 2019-03-14

**Authors:** Michael D Ballentine, Elizabeth G Embry, Marco A Garcia, Lawrence J Hill

**Affiliations:** 1 1906 College Heights Blvd., Western Kentucky University, Bowling Green, KY, 42101, USA

**Keywords:** catalyst, ionic liquid, methylene blue, platinum, semiconductor nanorod

## Abstract

The current study investigates whether metal deposition onto an existing nanorod can be carried out using an ionic liquid, and the effect this has on catalytic performance. Platinum, gold, and silver nanoparticles were deposited onto CdSe@CdS (core@shell) nanorods from metal salts in an ionic liquid (1-butyl-3-methylimidazolium bis(trifluoromethylsulfonyl)imide) without additional surfactants or reducing agents. Photocatalytic dye degradation experiments showed that catalysts with platinum particles deposited using the ionic liquid out-performed similar materials synthesized using organic solvents and ligands. We concluded that metal particles can be deposited onto well-defined semiconductor nanorods using ionic liquids and metal salts without the need for additional reagents, and the deposited particles did not cause significant aggregation even when these materials were taken into organic media. It is possible that a broad range of metal/semiconductor heterostructured particles can be prepared using the methods reported here.

## Introduction

Core@shell semiconductor nanorods with attached noble metal particles have been widely studied as photocatalysts, and any improvement on the synthesis of these materials has potential to impact a number of applications [1–4]. Deposition of metal nanoparticles onto the nanorod surface competes with homogeneous nucleation of the metal nanoparticles, with the size and number of metal nanoparticles attached to each nanorod controlled by lattice matching of the materials and synthesis conditions [5]. Well-defined nanorod substrates are synthesized using organic ligands to direct crystal growth [6], and these surface-bound ligands often play an important role in charge transfer at the particle/solvent interface [7–11]. However, these stabilizing ligands also insulate the underlying nanoparticle surface which complicates using these materials in device applications where the particles are coated onto a substrate [12]. A key feature of these well-defined nanorod systems is control of particle size and uniformity at small length scales; nanorods having at least two dimensions smaller than 10 nm are commonly reported with small deviations in the distribution of nanorod lengths.

Another widely investigated approach to nanoparticle synthesis uses ionic liquids as solvents for the synthesis of nanoparticles without the need for additional stabilizing surfactants [13–17]. The low surface tension of ionic liquids causes high nucleation rates and allows for the synthesis of small nanoparticles with minimal Ostwald ripening [17]. Further, many ionic liquids consist of a large anion with diffuse negative charge; this lack of strongly binding anionic ligands increases the surface availability of nanoparticles synthesized in ionic liquids. These so called “naked nanoparticles” are stabilized in ionic liquids both electrostatically and sterically through reversible interactions with the ionic liquid solvent [15], and do not suffer from performance limitations associated with strongly-bound organic ligands. In principle, it should be possible to prepare metal/semiconductor nanoparticle catalysts on sub-10 nm length scales using ionic liquids and benefit from increased performance due to the absence of strongly bound surface ligands. A literature search for using ionic liquids in deposition of metal particles onto semiconductor nanoparticles afforded many references involving deposition of nanoparticles onto graphene and carbon nanotubes [18–24]. CdS nanorods with average diameters below 10 nm have been synthesized by Rao et al. in ionic liquids using trioctylphosphine oxide and ethylenediamine for particle formation and stabilization [25]. However, to our knowledge, no one has explored metal deposition onto these nanorods and there are no examples using ionic liquids for metal deposition onto chalcogenide nanorods with uniform lengths and diameters smaller than 10 nm. This is important for the field of chalcogenide nanomaterials where 1D (nanorod) and 0D (quantum dot) materials are interfaced with metals on sub-10 nm length scales to facilitate charge transfer and photocatalysis [26].

**Scheme 1:**
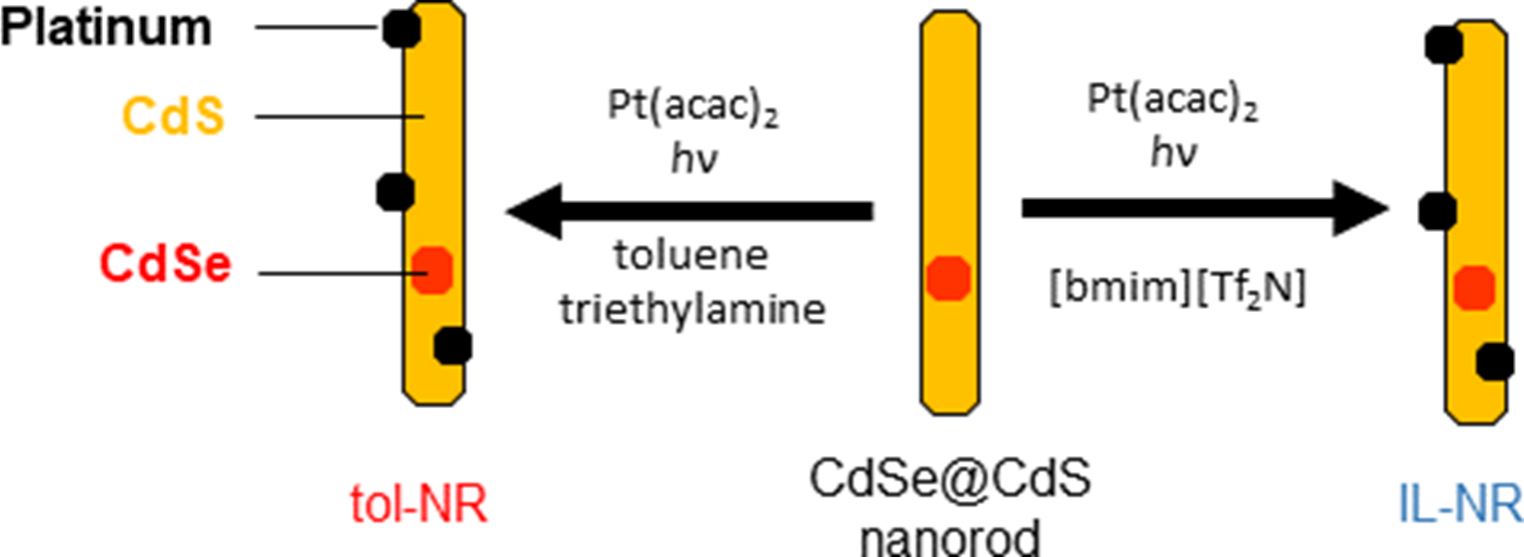
Platinum deposition onto CdSe@CdS nanorods.

Herein, we demonstrate that an ionic liquid may be used to deposit nanoscopic noble metal particles onto a well-defined semiconductor nanorod substrate with diameters less than 10 nm. We found that photodeposition of platinum onto CdSe@CdS (core@shell) nanorods proceeded readily from Pt(acac)_2_ in the ionic liquid 1-butyl-3-methylimidazolium bis(trifluoromethylsulfonyl)imide ([bmim][Tf_2_N]) without needing to add any additional reagents. We also deposited platinum nanoparticle cocatalysts onto CdSe@CdS nanorods using a traditional organic system (toluene/triethylamine) as shown in Scheme 1, and the two catalysts prepared in different solvents had remarkably similar morphologies as determined by analysis of TEM images. Inductively coupled plasma-atomic emission spectroscopy (ICP-AES) analysis of the elemental ratios in the samples confirmed the similarity of these materials, and we compared photocatalytic performance of these two samples for degradation of methylene blue. This hybrid approach using traditional organic ligands for the synthesis of well-defined nanorods followed by [bmim][Tf_2_N] for metal deposition was found to be applicable to other noble metals in addition to platinum. Gold and silver nanoparticles were also deposited onto CdSe@CdS nanorods without the need for additional surfactants or reducing agents.

## Results and Discussion

CdSe quantum dots and CdSe@CdS nanorods were synthesized using cadmium oxide, octadecylphosphonic acid, hexylphosphonic acid, trioctylphosphine, and trioctylphosphine oxide based on methods reported by Manna et al. and expanded upon by Pyun et al. [27–28]. Our CdSe quantum dots had an average diameter of 2.4 nm as determined using the correlation of particle diameter with the wavelength of the low energy absorbance peak (λ_max_ = 509 nm) [29]. The CdSe@CdS nanorods had an average length of 50 ± 10 nm and an average diameter of 4 ± 1 nm as determined by TEM imaging, with a sample size of at least 100 particles in each case.

**Figure 1:**
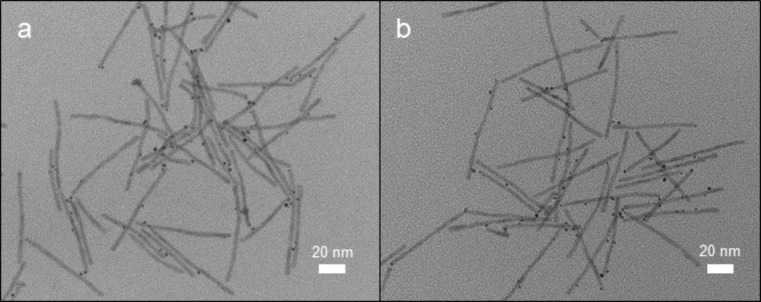
TEM images of Pt-decorated CdSe@CdS nanorods. a) Product of platinum deposition reaction conducted in toluene/triethylamine (tol-NR). b) Product of platinum deposition reaction conducted in [bmim][Tf_2_N] (IL-NR).

The obtained nanorods are capped with tightly bound phosphonic acid ligands bearing long hydrophobic alkyl chains. Rather than exchanging these ligands to allow these nanorods to be dispersed in polar media, platinum nanoparticles were deposited onto these CdSe@CdS nanorods in hydrophobic media based on the photochemical process reported by Alivisatos and coworkers [30], with the liquid component consisting of either toluene/triethylamine or [bmim][Tf_2_N] (Scheme 1). We chose [bmim][Tf_2_N] as the ionic liquid due to its facile synthesis and the hydrophobicity imparted by the fluorinated anion. For metal deposition in organic media (tol-NR, Figure 1a), CdSe@CdS nanorods were dispersed in toluene containing triethylamine and Pt(acac)_2_ followed by irradiation using a xenon arc lamp. For metal deposition using the ionic liquid, (IL-NR, Figure 1b), CdSe@CdS nanorods were dispersed in [bmim][Tf_2_N] containing Pt(acac)_2_ without addition of triethylamine as a sacrificial reducing agent, followed by irradiation using a xenon arc lamp. Nanorod powders were difficult to disperse directly in [bmim][Tf_2_N], so the nanorods were dispersed in a small amount of chloroform prior to mixing with the ionic liquid. Complete evaporation of the chloroform then afforded well-dispersed nanorods in the ionic liquid. The mass of the nanorod powder, Pt(acac)_2_, and the ionic liquid were each measured prior to dispersing the nanorods in chloroform and combining with the ionic liquid; complete removal of the chloroform was confirmed by taking the mass of the nanorod dispersion in [bmim][Tf_2_N] after evaporation of the chloroform under reduced pressure.

Platinum deposition under both solvent conditions initially afforded a mixture of platinum-decorated nanorods and additional platinum nanoparticles that were not attached to CdSe@CdS nanorods. For the platinum deposition conducted in toluene, the unattached platinum particles were removed by several dissolution and precipitation cycles using toluene and ethanol. For the platinum deposition conducted in [bmim][Tf_2_N], the platinum decorated nanorods were extracted into toluene from the ionic liquid prior to purification by centrifugation. The final pellets obtained were dispersed in toluene for storage and subsequent experiments regardless of the solvent used for the platinum deposition reaction. TEM images of both products are shown in Figure 1.

Although we observed that triethylamine was necessary for deposition of platinum particles onto CdSe@CdS nanorods dispersed in toluene, we found that platinum deposition onto the same nanorods occurred readily in [bmim][Tf_2_N] without needing to add a sacrificial reducing agent. The chemical species oxidized during platinum reduction and deposition onto these nanorods in [bmim][Tf_2_N] is not known at this time, though others have observed similar results. CoPt nanoparticles were previously formed in [bmim][Tf_2_N] using Pt(acac)_2_ and an ammonium bromide [31], and a mechanism involving the acetylacetonate ligands was suggested in a subsequent review article [15]. Additionally, a carbene mechanism involving the imidazolium cation could also contribute to the reduction mechanism [32], as proposed for Pd nanoparticles formed sonochemically from Pd(OAc)_2_ in imidazolium ionic liquids containing acetate ligands [33]. Au(III) also slowly reduces to Au(0) in imidazolium ionic liquids, and this process can be facilitated by addition of cellulose to the reaction mixture as a mild reducing agent [34]. Thus, there is precedent for using pure imidazolium ionic liquids to facilitate reduction of metal salts to zero valent metal particles.

The heterostructured nanorods we obtained from the ionic liquid and the particles obtained from toluene/triethylamine had remarkably similar morphologies as determined by TEM imaging (Figure 1). Quantification of the TEM images confirmed that both samples had similarly sized platinum nanoparticles and a similar number of platinum particles per nanorod (Table 1, a minimum of 100 particles were counted/sized in each case). Using this information, we estimated comparable platinum to cadmium molar ratios based on the size of the CdS and Pt particles obtained from TEM images assuming bulk density for the cadmium sulfide and platinum particles. The concentration of cadmium and platinum determined using inductively coupled plasma-atomic emission spectroscopy (ICP-AES) analysis provided complimentary evidence that both samples had similar elemental ratios of platinum to cadmium (Table 1). When taken with the comparable morphologies observed by TEM imaging, the similar platinum to cadmium ratio in both samples is consistent with removal of the majority of free platinum particles from the heterostructured nanoparticles.

**Table 1:** Comparison of sample composition determined by TEM and ICP-AES.

Sample name	Platinum particle diameter (nm)	Platinum particles per nanorod	Cd/Pt molar ratio (estimated)	Cd/Pt molar ratio (from ICP-AES)	Weight % Pt per inorganic particle

tol-NR	2.2 ± 0.1	2.4 ± 0.6	16.10	11.13	10.8
IL-NR	2.3 ± 0.2	2.4 ± 0.5	16.02	11.65	10.4

**Figure 2:**
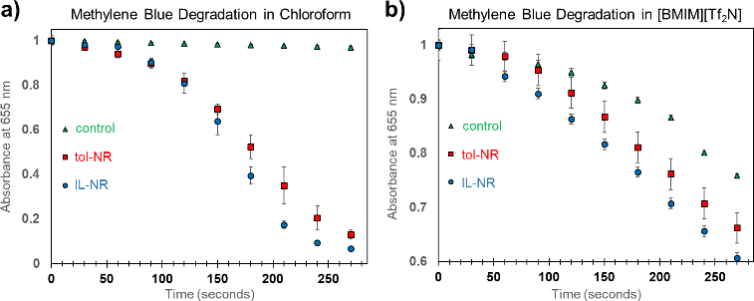
Comparison of photocatalytic activity for tol-NR and IL-NR. a) Dye degradation experiments conducted in chloroform. b) Dye degradation experiments conducted in [bmim][Tf_2_N]. The control in both cases shows dye degradation without a catalyst present. All data points have error bars representing the standard deviation of three trials per data point.

With two nearly identical samples in hand, we were able to compare the performance of two catalysts where the only notable difference was the synthetic history of the sample (Figure 2). We used photocatalytic degradation of methylene blue to compare catalyst performance since this is a well-studied system that has been used to mimic two-electron processes such as the hydrogen evolution reaction [35]. Both nanomaterials were stored in toluene prior to methylene blue degradation experiments to minimize any effect from residual ionic liquid solvent in the IL-NR sample, and both catalysts were prepared from a single batch of CdSe@CdS nanorods to eliminate any differences in nanorod substrates from our study. Thus, the only significant difference between the samples at the time of dye degradation experiments was whether the platinum particles were deposited in [bmim][Tf_2_N] or toluene/triethylamine.

A known amount of the nanoparticle catalyst was combined with methylene blue and chloroform for photocatalytic dye degradation experiments (Figure 2a), based on the concentration of cadmium and platinum in the toluene dispersions obtained from ICP-AES. Control experiments were also conducted to observe the dye degradation rate without catalyst present. The samples were irradiated with visible light and dye degradation was monitored using a UV–vis spectrometer to track the disappearance of the characteristic absorbance peak at 655 nm. Although both catalysts were stored in toluene after deposition of platinum onto the nanorods and the dye degradation experiments were conducted in chloroform, the catalysts decorated with platinum in [bmim][Tf_2_N] showed a measurably increased dye degradation rate compared to the sample prepared in toluene/triethylamine. Error bars corresponding to the standard deviation values of these experiments show that the obtained results are reproducible for these materials (Figure 2). Note that the dye degradation experiments (control, tol-NR, and IL-NR) were each conducted once before duplicating and then triplicating the entire set of experiments to increase confidence in our results. We also conducted dye degradation experiments in the ionic liquid [bmim][Tf_2_N] to rule out any possible effects from the ionic liquid itself (Figure 2b). Although dye degradation occurred rapidly in the ionic liquid even without catalyst present, we still observed faster dye degradation for the catalyst prepared using the ionic liquid compared to the catalyst prepared in toluene.

These heterostructured nanorod photocatalysts bleach methylene blue by donating electrons from platinum cocatalyst sites to the dye [36]. Thus, the enhanced catalytic activity of samples prepared in the ionic liquid versus toluene/triethylamine may be due to decreased organic content strongly associated with the platinum nanoparticle catalytic sites. This is consistent with previous reports that showed triethylamine binding to the surface of platinum nanoparticles [37], and the known improvement in catalyst performance for metal particles synthesized in ionic liquids [13]. However, the increase in dye degradation rate observed for IL-NR compared to tol-NR, though statistically significant and reproducible, was narrow. This modest improvement in dye degradation is consistent with previous findings that hole removal rate at the nanorod surface, rather than electron removal rate at the platinum surface, has a larger impact on the catalytic efficiency for these metal-semiconductor hybrid nanorods [38]. Because alkyl phosphonic acid ligands cap the nanorod surface of both materials (IL-NR and tol-NR), both materials may react at similar rates with the solvent to neutralize accumulated positive charges in the catalyst.

**Figure 3:**
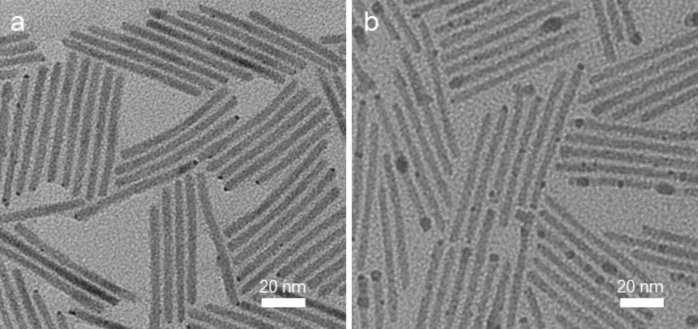
TEM images of metal-decorated CdSe@CdS nanorods prepared using [bmim][Tf_2_N] and metal precursor. a) Au-decorated nanorods obtained from AuCl_3_. b) Ag-decorated nanorods obtained from AgNO_3_.

We then investigated reduction of gold and silver in the presence of CdSe@CdS nanorods using [bmim][Tf_2_N] without any additional surfactants or reducing agents to determine if this ionic liquid was generally useful for deposition of other metals (Figure 3). For gold deposition, CdSe@CdS nanorods were dispersed in [bmim][Tf_2_N] as described above, AuCl_3_ dissolved in [bmim][Tf_2_N] was added, and the mixture was heated in a 50 °C water bath for 10 minutes. The nanorod product was then extracted into toluene and a TEM sample was prepared without further purification (Figure 3a). We observed that Au particles with diameters of 2 ± 1 nm formed preferentially on the termini of semiconductor nanorods, and the morphologies of these gold-decorated nanorods were similar to those previously observed using organic solvents and ligands [39–40]. We were also able to deposit silver nanoparticles onto CdSe@CdS nanorods using only [bmim][Tf_2_N] and AgNO_3_ at 50 °C (Figure 3b). Due to the lower contrast in electron microscopy of Ag compared to Au, silver deposition was conducted at higher precursor concentration than gold deposition experiments to increase the particle size and facilitate imaging of the attached particles; as a result, silver deposition afforded a broader range of particle diameters (4 ± 2 nm) and less well-defined particle morphologies than those observed using gold.

## Conclusion

In conclusion, we have demonstrated that deposition of noble metal nanoparticles onto CdSe@CdS nanorods is possible using the ionic liquid [bmim][Tf_2_N] without the need for additional surfactants or reducing agents. These procedures resulted in heterostructured particles with similar morphology and elemental composition compared to particles synthesized using traditional organic solvents and ligands. The performance of the resulting materials with metal cocatalysts deposited in [bmim][Tf_2_N] showed only modest improvement over materials prepared in conventional solvents, which is attributed to the fact that the semiconductor nanorod substrate was still capped with insulating organic ligands in both samples. The methods reported herein could be used to achieve a multistep synthesis of metal-decorated core@shell particles using only ionic liquids to determine if the increased surface availability will benefit catalytic performance. This approach using an ionic liquid for deposition of metal particles onto semiconductor nanoparticles may enable greener synthetic approaches involving recycling the ionic liquid solvent, and the large liquid range of the ionic liquid also allows studying of photocatalysis over a broad range of temperatures.

## Supporting Information

File 1 Synthetic procedures and raw data for methylene blue degradation experiments.
