# Supplementary material for: Deposition of metal particles onto semiconductor nanorods using an ionic liquid
Source: Beilstein J Nanotechnol. 2019 Mar 14;10:718–24. doi: 10.3762/bjnano.10.71 (PMC6423590; doi:10.3762/bjnano.10.71)
Supplement: File 1 — Synthetic procedures and raw data for methylene blue degradation experiments. [file Beilstein_J_Nanotechnol-10-718-s001.pdf]

BEILSTEIN JOURNAL OF NANOTECHNOLOGY

Supporting Information

for

Deposition of metal particles onto semiconductor nanorods using an ionic liquid

Michael D. Ballentine, Elizabeth G. Embry, Marco A. Garcia and Lawrence J. Hill

Beilstein J. Nanotechnol. **2019,** *10,* 718–724. doi:10.3762/bjnano.10.71

Synthetic procedures and raw data for methylene blue degradation experiments

License and Terms: This is a supporting information file under the terms of the Creative Commons Attribution License (http://creativecommons.org/licenses/by/4.0). Please note that the reuse, redistribution and reproduction in particular requires that the authors and source are credited.
The license is subject to the *Beilstein Journal of Nanotechnology* terms and conditions: (https://www.beilstein-journals.org/bjnano)

Materials and equipment

The ionic liquid 1-butyl-3-methylimidazolium bis(trifluoromethylsulfonyl)imide ([bmim][Tf_2N]) was prepared by metathesis of 1-butyl-3-methylimidazolium chloride ([bmim][Cl]) with lithium bis(trifluoromethylsulfonylimide)[1]. All other chemicals were commercially available and used as received. Image analysis was performed using ImageJ software (Rasband, W.S., National Institutes of Health, http://rsb.info.nih.gov/ij/, 1997-2007) from bright field TEM images by sizing a minimum of 100 particles.

TEM samples were imaged using a JEOL 1400Plus transmission electron microscope using both the 100kV and 120kV voltages. Copper 200 mesh grids with a carbon coating were used as a sample holder to get the images. The carbon was from EMS Cat #70200, sharpened to about 4 mm with a #12040 carbon rod sharpener from E. Fullam. ICP-AES experiments were conducted using a ThermoScientific ICAP 6500 series ICP-OES. UV-Vis spectroscopy was performed using a UV-2600 UV-VIS Spectrophotometer by Shimadzu. A 0.5 mL quartz cuvette was used as a sample container and scanned from 700 nm to 300 nm with 0.5 nm increments. A xenon arc lamp by Newport (model 69907, 300 watts) was used for platinum deposition experiments.

Synthesis of CdSe quantum dots and CdSe@CdS nanorods

CdSe quantum dots and CdSe@CdS nanorods were synthesized as previously reported [2], based on the work of Manna et al. [3].

Synthesis of platinum decorated nanorods

tol-NR. CdSe@CdS nanorods (20 mg dried powder) were dispersed in toluene (17.2 mL) containing triethylamine (2.8 mL) and $Pt(acac)_2$ (60 mg) in a 50 mL quartz round bottom flask with stir bar. The flask was sealed with a septum and irradiated with a xenon arc lamp placed directly in contact with the outer wall of the flask at full power (300 watts) for four hours with continuous stirring to afford a black dispersion. The crude product was divided between two 50 mL centrifuge tubes and each tube was diluted to 50 mL with ethanol. Centrifugation at 2500 rpm for 12 minutes afforded a black pellet which was retained and a clear supernatant which was discarded. The pellets were dispersed in toluene and precipitated with ethanol followed by centrifugation as above. The pellets obtained after four centrifugation cycles were dispersed in toluene and stored in a sealed vial. This toluene

dispersion was evaluated by TEM and ICP analysis prior to dye degradation experiments as described in the main article.

IL-NR. [bmim][Tf_2N] (approximately 6 mL) was added to a tared vial on a balance to determine the exact mass added. CdSe@CdS nanorods dispersed (6 mg dried powder) in chloroform (2 mL) were added to the vial, and the liquids were combined by vortexing and sonication. Chloroform was then removed under reduced pressure, and the mass of the nanorod dispersion in [bmim][Tf_2N] was measured to confirm removal of the chloroform. The nanorod dispersion was transferred to a quartz round bottom flask containing $Pt(acac)_2$ (18 mg) and a stir bar. The flask was sealed with a septum and irradiated with a xenon arc lamp at 300 W for four hours with continuous stirring to afford a black dispersion. Note that toluene and [bmim][Tf_2N] are partially miscible such that they form two phases when the volume of toluene exceeds that of the ionic liquid, and the platinum decorated nanorods prepared in the ionic liquid strongly favored the toluene phase in liquid/liquid extractions. Therefore, the black dispersion was extracted twice with toluene to afford a dispersion of platinum decorated nanorods in toluene. This dispersion was subjected to five centrifugation cycles as described above for tol-NR, and the final product was stored in toluene prior to TEM and ICP analysis.

CdSe@CdS nanorod stock solutions for gold and silver deposition reactions

[bmim][Tf_2N] (approximately 5 mL) was added to a tared vial on a balance to determine the exact mass added. CdSe@CdS nanorods (10 mg dried powder) dispersed in chloroform (2 mL) were added to the vial and the liquids were combined by vortexing and sonication. Chloroform was then removed under reduced pressure, and the mass of the nanorod dispersion in [bmim][Tf_2N] was measured to confirm removal of the chloroform. The nanorod dispersion was sparged with argon for 15 minutes and sealed with a septum.

Synthesis of Au-decorated nanorods

A solution of $AuCl_3$ (10 mg) in [bmim][Tf_2N] (5 mL) was prepared under argon. The nanorod stock solution in [bmim][Tf_2N] (0.5 mL) and $AuCl_3$ solution in [bmim][Tf_2N] (0.5 mL) were combined in a clean vial with stir bar under argon and the mixture was lowered into a 50 °C water bath with stirring for 10 minutes. The nanorod product was then extracted into toluene (2 mL) and a TEM sample was prepared without further purification.

Synthesis of Ag-decorated nanorods

A solution of $AgNO_3$ (10 mg) in [bmim][Tf_2N] (0.5 mL) was combined with the nanorod stock solution in [bmim][Tf_2N] (0.5 mL) in a clean vial with stir bar in air, and the mixture was lowered into a 50 °C water bath with stirring for 10 minutes. The nanorod product was then extracted into toluene (2 mL) and a TEM sample was prepared without further purification.

Methylene blue degradation experiments

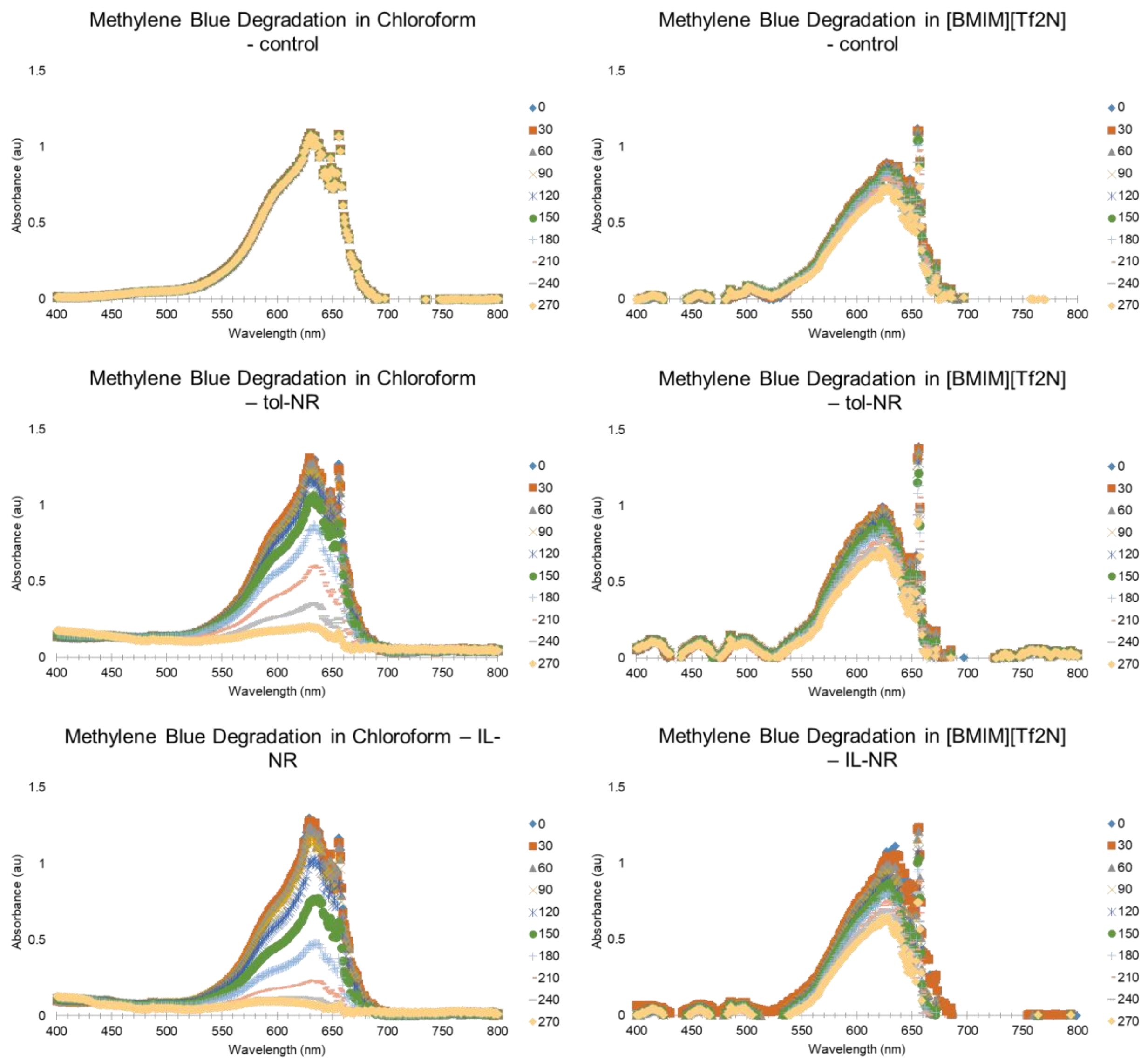

Figure S1: Representative absorbance data taken to track degradation of methylene blue.

Methylene blue degradation experiments were conducted by irradiating the sample with visible light from a SOLA SE II light engine (Lumencor) configured with a liquid light guide (120W). Due to the increased rate of dye degradation without catalyst present in [bmim][Tf_2N], degradation experiments in the ionic liquid were conducted at 5% intensity of incident light relative to degradation experiments conducted in chloroform. Representative UV-vis data taken during methylene blue degradation experiments are shown in Figure S1.

References

1. Bruce, D. W., Gao, Y., Canongia Lopes, J. N., Shimizu, K. and Slattery, J. M., *Chem. - Eur. J.*, **2016**, *22*, 16113-16123.
2. Hill, L. J., Bull, M. M., Sung, Y., Simmonds, A. G., Dirlam, P. T., Richey, N. E., DeRosa, S. E., Shim, I.-B., Guin, D., Costanzo, P. J., Pinna, N., Willinger, M.-G., Vogel, W., Char, K. and Pyun, J., *ACS Nano*, **2012**, *6*, 8632-8645.
3. Carbone, L., Nobile, C., De Giorgi, M., Sala, F. D., Morello, G., Pompa, P., Hytch, M., Snoeck, E., Fiore, A., Franchini, I. R., Nadasan, M., Silvestre, A. F., Chiodo, L., Kudera, S., Cingolani, R., Krahne, R. and Manna, L., *Nano Lett.*, **2007**, *7*, 2942-2950.
